# Comparative Analysis of Vascular Calcification Risk Factors in Pre-Hemodialysis and Prevalent Hemodialysis Adult Patients: Insights into Calcification Biomarker Associations and Implications for Intervention Strategies in Chronic Kidney Disease

**DOI:** 10.3390/diagnostics14080824

**Published:** 2024-04-17

**Authors:** Marko Petrović, Voin Brković, Marko Baralić, Ivko Marić, Nenad Petković, Sanja Stanković, Nataša Lalić, Dejana Stanisavljević, Ljubica Đukanović, Višnja Ležaić

**Affiliations:** 1 Department of Nephrology, University Clinical Centre of Serbia, 11000 Belgrade, Serbia; markofbkbgd@gmail.com (M.P.); baralicmarko@yahoo.com (M.B.); 2 Faculty of Medicine, University of Belgrade, 11000 Belgrade, Serbialjubicadjukanovic@yahoo.com (L.Đ.); 3 Special Hospital for Internal Diseases, 11550 Lazarevac, Serbia; 4 Fresenius Medical Care Dialysis Center, 76230 Šamac, Bosnia and Herzegovina; 5 Centre for Medical Biochemistry, University Clinical Centre of Serbia, 11000 Belgrade, Serbia; sanjast2013@gmail.com; 6 Faculty of Medical Sciences, University of Kragujevac, 34000 Kragujevac, Serbia; 7 Uromedica Polyclinic Belgrade, 11000 Belgrade, Serbia

**Keywords:** chronic kidney disease, vascular calcifications, FGF 23, fetuin-A, sclerostin, iPTH, Adragao score

## Abstract

This retrospective study aimed to compare risk factors for vascular calcification (VC) between pre-hemodialysis (HD) and prevalent HD adult patients while investigating associations with calcification biomarkers. Baseline data from 30 pre-HD and 85 HD patients were analyzed, including iPTH, vitamin D, FGF 23, fetuin-A, sclerostin, and VC scores (Adragao method). Prevalence of VC was similar in both groups, but HD patients had more frequent VC scores ≥ 6. Pre-HD patients were older, with higher prevalence of hypertension and less frequent use of calcium phosphate binders. Both groups showed similar patterns of hyperphosphatemia, low vitamin D, and iPTH. Fetuin-A and sclerostin levels were higher in pre-HD, while FGF 23 was elevated in HD patients. Higher VC risk in pre-HD patients was associated with male gender, older age, lower fetuin-A and higher sclerostin, lower ferritin, and no vitamin D treatment, while in HD patients with higher sclerostin, FGF 23 and urea, and lower iPTH. Conclusion: Biomarkers could be measurable indicators of biological processes underlying VC in CKD patients that may serve as a potential guide for considering personalized therapeutic approaches. Further studies are needed to elucidate the underlying pathways.

## 1. Introduction

Chronic kidney disease (CKD) represents a significant global health burden, affecting millions of individuals worldwide. With its rising prevalence, CKD poses substantial challenges to healthcare systems globally and is associated with increased morbidity, mortality, and healthcare costs [1]. Vascular calcification (VC) stands as a hallmark complication of CKD, with its prevalence increasing across CKD stages, culminating in its highest incidence in end-stage kidney disease (ESKD) patients [2]. Evidence suggests that CKD patients having VC from stage 3 to the beginning of dialysis treatment have a more rapid progression of calcification during dialysis [3,4]. The clinical consequences of VC in CKD are profound, significantly contributing to cardiovascular morbidity and mortality [2,5].

There is growing evidence that patients with CKD are prone to VC due to disturbed homeostatic mechanisms. Existing literature highlights the interplay of various risk factors, encompassing traditional elements such as age, hypertension, and diabetes, along with emerging contributors like oxidative stress, mineral metabolism disturbances, and renal bone disease [6]. Additionally, imbalances in circulating biomarkers including intact parathyroid hormone (iPTH), fibroblast growth factor 23 (FGF 23), and sclerostin that promotes or Fetuin-A that inhibits VC processes have emerged as potential markers of VC susceptibility of CKD patients [4,6,7].

Emerging insights highlight the active role of vascular smooth muscle cells (VSMCs) transitioning into osteoblast-like cells in VC pathogenesis [7]. However, the molecular underpinnings, especially the interplay of calcification promoters and inhibitors, remain inconsistent and an evolving area of research [6,8].

While earlier studies focused on establishing the involvement of biomarkers in the pathophysiology of VC in CKD patients, contemporary therapeutic approaches now aim to leverage biomarkers for the prevention and treatment of VC in this population [3,9,10,11,12]. Despite some promising results, they are not yet in use for VC treatment. So far, studies examining the dynamics of VC biomarker changes during the progression from CKD to the dialysis phase are lacking. Understanding how these biomarkers evolve in advanced CKD is crucial for identifying specific targets for interventions aimed at preventing or mitigating VC.

In this context, the comparative analysis between pre-hemodialysis and prevalent hemodialysis (HD) patients assumes utmost importance. As CKD progresses to ESKD requiring dialysis, the risk and severity of VC may undergo significant changes, necessitating tailored therapeutic interventions. However, to date, there is a paucity of studies directly comparing the clinical and biochemical profiles of VC between these two patient groups. Furthermore, it is vital to note that VC treatment primarily relies on controlling various risk factors, emphasizing the ongoing need to discover new therapeutic targets to mitigate the adverse consequences of VC. In the available literature, there are no papers that simultaneously analyze the change in biomarkers in patients with advanced CKD and those treated with dialysis. Therefore, this cross-sectional study was undertaken to compare clinical and biochemical profiles, with special attention to changes in some biomarkers between two groups of patients with VC: the pre-hemodialysis (pre-HD) and prevalent HD patients. By elucidating the differences and similarities in VC between these two patient cohorts, the current study seeks to inform clinical practice and guide future research directions aimed at improving outcomes in CKD patients.

## 2. Materials and Methods

**Figure 1:**
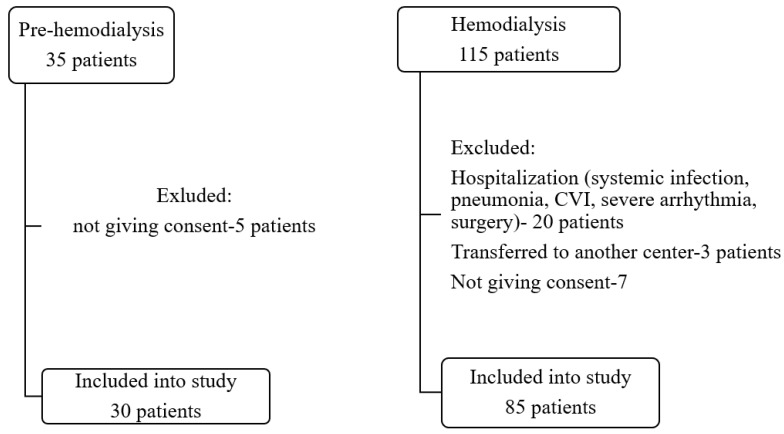
Flowchart patient selection.

This cross-sectional study involved 30 pre-hemodialysis adult patients (pre-HD group: mean age 66.7, gender: 19 males) and 85 adults on hemodialysis (HD group: mean age 57.7, gender: 40 males) selected from the pool of patients monitored in outpatient departments or treated by chronic HD in three nephrology departments presented in the flow chart (Figure 1).

Patients were enrolled if they: (1) had eGFR < 15 mL/min/1.73 m^2^ and were regularly controlled by a nephrologist in the above-mentioned departments (pre-HD group) or had been on HD for at least 6 months (HD group); (2) agreed to participate in the study, which was approved by the institutional review board; (3) did not have acute cardiovascular complications during the 6 months preceding entry into the study; (4) had no hemodynamically significant lower extremity artery occlusive disease; (5) did not have uncontrolled blood pressure.

The local Ethics Committee evaluated and approved the study protocol (decision No 1690/21, 9 June 2015) and all patients provided written informed consent.

Standard bicarbonate HD sessions lasted 12 h weekly. Dialysate calcium (dCa) was individualized to meet the specific requirements of each patient by optimizing treatment to achieve the target values of serum calcium, phosphate, iPTH, and alkaline phosphatase levels recommended by the KDIGO guideline [13]. Calcium-based phosphate binders, alphacalcidol, or calcitriol were mostly used in studied patients. Variables of interest taken from the patients’ records were: demographic (age, sex), underlying kidney disease, dialysis duration, systolic and diastolic blood pressures, previous history of cerebrovascular and cardiovascular diseases: coronary artery disease (CAD); congestive heart failure (CHF); peripheral vascular disease (PVD); parathyroidectomy due to secondary hyperparathyroidism and medications (antihypertensives, intestinal phosphate binders, Vitamin D, erythropoetin stimulating agents (ESA), iron treatment). Blood pressure (BP) measurement is part of a routine examination. BP was measured during hemodialysis every hour from the beginning of hemodialysis, and the value for statistical processing was expressed as the mean value of systolic and diastolic BP. In the group of pre-HD patients, BP was measured in the outpatient department, following the European Society of Hypertension—Cardiology guidelines [14], and expressed as the mean value of systolic and diastolic BP calculated from measured BP values during the last six months of control. Patients with BP ≥ 140/90 are classified as having high BP (counted as 1), and when the BP was below these values, it was marked with 0.

### 2.1. Biochemical Analyses

The routine biochemical analyses and measurement of VC biomarkers were done using standard techniques in the same laboratory. Routine laboratory analyses were performed before middle week HD in the HD patients or at regular controls in the outpatient department in the pre-HD patients. Also, serum samples for biomarkers were frozen at −80 degrees until analysis, which was done altogether at the same time. Laboratory analyses included measurement of serum urea, creatinine, uric acid, total protein, C-reactive protein (CRP), lipid profile, phosphate, calcium corrected for albumin, alkaline phosphatase, hematological parameters, iron status, and iPTH. Mean standard weekly Kt/V and urea reduction ratio (URR) were calculated for the HD group. We also measured serum levels of 25-(OH) Vitamin D, FGF 23, Fetuin-A, and sclerostin. An immunoradiometric assay was used to measure iPTH (enzyme-linked immunosorbent assay (ELISA) PTH, CIS bio International) and normal values are 11–62 pg/mL. A commercial chemiluminescent immunoassay (DiaSorin S.p.A., Saluggia, Italy) was used to determine serum 25-(OH) Vitamin D, and the measuring range is 7.6–147.8 ng/mL; values of 21–29 ng/mL are considered insufficient, and <20 ng/mL vitamin D as deficient [15]. Serum levels of sclerostin, FGF 23, and Fetuin-A were determined in duplicate by using commercially available ELISA kits (Elabscience, and Cusabio, Houston, TX, USA). The detection assay range for sclerostin, intact FGF 23, and Fetuin-A were 62.4–4000 pg/mL, 15–2000 pg/mL, and 9.375–600 ng/mL, respectively, with reported intra-assay precision of <10%.

### 2.2. Calcification Assessment

Vascular calcification (VC) in the iliac, femoral, radial and digital arteries in plain radiographic films of the pelvis and hands were evaluated by one person. A simple VC score was calculated as described by Adragao et al. [16]. Although there are more sophisticated methods for VC assessment; being aware that determining the Adragao score is a less sensitive measure, for practical reasons, we decided to use that method. The presence of linear calcifications in each section is counted as 1 and its absence as 0. The final score is the sum of all the sections, ranging from 0 to 8.

### 2.3. Statistical Analysis

Statistical analysis was performed with SPSS 21.0 (SPSS, Inc., Chicago, IL, USA). Continuous variables are presented as means with standard deviation (SD) for normally distributed variables and as median (inter-quartile range, IQR) for non-normally distributed variables. To achieve a more precise estimation, the values of Fetuin A, Sclerostin, FGF 23, and iPTH were transformed into logarithmic values. Categorical variables are presented as frequencies. Chi-square tests and Kruskal–Wallis one-way analysis were used to examine differences in various baseline variables between the groups of patients. A multivariable linear regression model including all significant variables in the univariate linear regression models (at a significance level of 0.1), as well as those predictors that are known to affect the dependent variable mentioned in the Methods Section, i.e., demographic data, standard laboratory analyses, Ln(FGF 23), Ln(iPTH), vitamin D, Ln(fetuin-A) and Ln(sclerostin), and VC score, was used to determine the independent association with VC score as the dependent variable. To avoid collinearity, several models were used. Two-sided *p*-values < 0.05 were considered significant.

## 3. Results

### 3.1. Study Population

Baseline characteristics for both studied groups are shown in Table 1. Comparatively, pre-HD patients were older and had a higher prevalence of HTZ, while HD patients were more inclined to receive calcium-based phosphate-lowering treatment. The primary causes of ESKD were comparable between the groups. The median HD vintage before inclusion in this study was 69.5 (125.5) months. Subtotal parathyroidectomy had been performed in 11 HD patients before their enrollment.

Among the 115 subjects under study, VC was detected in 46.6% of pre-HD patients and 57.6% of HD patients. Further analysis revealed a marked VC score ≥ 6 in one patient within the pre-HD group and 15 HD patients, representing a statistically significant difference (*p* = 0.030). Additionally, the highest Adragao score of 8 was observed in six HD patients and was not observed within the pre-HD patient group (Table 1).

### 3.2. Laboratory Analyses

Table 2 presents the basal laboratory analyses. In comparison to the pre-HD group, the HD group exhibited significantly higher serum concentrations of creatinine, potassium, and ferritin, while serum sodium was significantly lower. Mean serum levels of calcium, phosphate, intact parathormone (iPTH), and vitamin D fell within the normal range for all patients. Individual values revealed that two HD patients had mild hypercalcemia at 2.7 mmol/L. Also, phosphatemia out of the normal range (1.0–1.8 mmol/L) was observed in 33.3% of the pre-HD group and 35.3% of HD patients. Furthermore, 50% of pre-HD patients and 56.5% of those in the HD group had iPTH values below 100 pg/mL.

Vitamin D concentration below the lower limit of the reference range was found in 64% of pre-HD patients and 53.6% of those in the HD group. In terms of circulating biomarkers, serum levels of FGF 23 were notably lower (*p* < 0.001) in the pre-HD group, while serum Fetuin-A and sclerostin concentrations were significantly higher (*p* < 0.001) in comparison to the HD group.

Statistically significant differences obtained by comparing all clinical and biochemical parameters mentioned in the Methods Section, within and between groups, with and without VC, were shown in Table 3. Among pre-HD patients, those with VC were notably older, and a higher proportion were males. In both HD patient subgroups, there was more frequent use of phosphate binders compared to pre-HD patients. HD patients without VC had the highest mean phosphate values.

Moreover, patients without VC in both groups more often exhibited hyperphosphatemia (>1.9 mmol/L) than those without VC, with the difference approaching significance (*p* = 0.057). Furthermore, within the HD group, a significant difference was observed in phosphate distribution; more patients with VC had normal phosphate levels (*p* = 0.02), while more patients without VC had phosphate above the reference range (*p* = 0.005). Regardless of the presence or absence of VC, HD patients displayed higher FGF 23 levels and lower Fetuin-A concentrations compared to their pre-HD counterparts.

### 3.3. Predictors of Vascular Calcification in Studied Groups

Table 4 and Table 5 provide the predictors of VC across the patient groups. In the univariate linear regression analysis, demographic data (age, gender), diagnosis of cardiovascular diseases, serum urea, creatinine, uric acid, hemoglobin, ferritin, phosphate, calcium, alkaline phosphatase, biomarkers, phosphate binders, or vitamin D treatment are exhibited associations with VC.

Patient age, gender, serum sclerostin, fetuin-A, ferritin, and vitamin D treatment finally appear to be significant predictors of VC in the pre-HD group (Table 4). In other words, higher levels of serum sclerostin and patient age are associated with increased VC, while higher levels of serum fetuin-A are associated with decreased VC, all with moderate effect. Being female is also associated with decreased VC compared to being male. Also, higher levels of serum ferritin had a mild protective effect on VC. Receiving vitamin D treatment is associated with a reduction in VC, with a weak effect.

The multivariable linear regression analysis in the HD group (Table 5) highlighted that the higher levels of sclerostin and FGF 23 (both moderate effects), along with elevated urea (strong effect), are associated with increased VC. Meanwhile, higher levels of PTH are associated with a decrease in VC (strong effect).

## 4. Discussion

The prevalence of VC among patients with CKD has long been recognized as a complex and concerning phenomenon. The present study sought to compare the frequency and potential risk factors and biomarkers associated with VC in both pre-HD and prevalent HD patients. The key findings are as follows: (1) VC is present in substantial of the examined patients in both groups; (2) while the prevalence of VC remained comparable, the severity of VC differed notably, with the HD group displaying a more prominent VC score ≥ 6; (3) examined circulating biomarkers: iPTH, FGF 23, sclerostin, and Fetuin-A were associated with VC; (4) serum urea, age, gender, and treatment with vitamin D appear to be additionally associated with VC.

Our findings are in agreement with previous studies showing a significant prevalence of VC in the pre-HD and HD groups. While the prevalence of VC remained comparable between the groups, i.e., 46.6% of pre-HD patients and 57.6% of HD patients, its noteworthy divergence emerged in the severity of VC. The HD group exhibited a more pronounced VC score of ≥6. Prior research has shown that rates of VC measured by the Adragao score remain similar among advanced CKD patients, reaching up to 57% in CKD stages 4–5. However, the prevalence increases to as high as 75% among dialysis patients [16,17,18]. Recent studies have shown that dialysis not only contributes to the progression of VC but also triggers its onset [19]. The similar prevalence of VC in both pre-HD and HD groups raises questions about whether there are shared underlying risk factors or if the progression of VC is influenced by different factors in each group.

### 4.1. Association of Biomarkers and Vascular Calcification in Pre-HD and HD Group of Patients

The investigation of various biomarkers in the present study revealed their dynamic involvement in VC in pre-HD and HD. This change is marked by a shift in the balance between inhibitors and promoters of VC, favoring the latter.

One important VC inhibitor, fetuin-A, is known for its ability to mitigate ectopic calcification in CKD and ESKD kidney disease. By impeding calcium phosphate precipitation, fetuin-A serves as a guardian, protecting human vascular smooth muscle cells from damage [20]. The progressive decline in serum fetuin-A levels from CKD stage 2 to 5 reaches its lowest point in dialysis patients. Discrepancies exist in the literature on the role of fetuin-A in VC development. While some studies report a correlation between persistently low serum fetuin-A levels and increased arterial and valvular calcification scores in patients with CKD and ESRD [21], others do not confirm such an association [22]. Although the values were within normal laboratory limits, our findings revealed significantly higher concentrations of fetuin-A in the pre-HD group than in the HD group, which is consistent with prior research. Regardless of the higher concentration, an inverse relationship with VC was observed, indicating that lower levels of fetuin-A correlate with more frequent VC. This reversal challenges the conventional perception of fetuin-A as a straightforward inhibitor and prompts the consideration of other factors or interactions that may modulate its effectiveness in preventing VC during the pre-HD phase.

Another biomarker associated with VC in pre-HD patients is sclerostin levels. Although its concentration exhibited a significant increase in the pre-HD group compared with HD patients, a notable positive association between VC and sclerostin was identified. This finding suggests that the influence of sclerostin may surpass the impact of higher fetuin-A concentrations in promoting VC at this stage of CKD. The significance of sclerostin in promoting VC appears to be noteworthy, even with a seemingly modest elevation in its concentration.

Sclerostin, a glycoprotein synthesized by osteocytes, primarily operates by inhibiting the canonical Wnt–β-catenin pathway and has the potential to attenuate bone formation and mineralization. While it is well established that sclerostin has extraskeletal functions, particularly in various vascular disorders, the precise mechanism by which it influences the VC process remains controversial. Some authors have reported positive associations, suggesting the promotion of calcification [23], but others have found negative associations, indicating an inhibitory effect [24].

In the HD group, our findings revealed a positive association between VC, sclerostin, and FGF 23, whereas iPTH was negatively associated. These results align with the existing literature, highlighting the roles of sclerostin and FGF 23 as VC promoters.

FGF 23, primarily produced by osteocytes in the bone, exerts inhibitory effects on 1,25(OH)2D and iPTH production, playing a crucial role in phosphate regulation by suppressing intestinal phosphate absorption and reabsorption in the proximal tubules. In CKD, elevated FGF 23 levels serve as a compensatory response to the reduced ability of the kidneys to excrete phosphate. However, persistently high FGF 23 levels can lead to mineral imbalances and bone abnormalities, contributing to mineral bone disease [25]. Conflicting results on the association between elevated FGF 23 levels and VC in CKD have been reported [26,27,28,29]. Clinical studies of HD and CKD stages 2–5 patients indicate a positive association of higher FGF 23 levels and increased VC, as we have found [25,26,27,28]. In contrast, Scialla et al. reported that a much larger cohort of 1501 patients with CKD stages 2–4 from the CRIC (Chronic Renal Insufficiency Cohort) study did not show such a relationship between FGF 23 levels and the prevalence of coronary artery calcification [29].

The third biomarker, iPTH level, was negatively associated with VC in our HD group, indicating that the lower the iPTH level, the higher the risk of developing VC. Based on low iPTH levels, we suspected that 42% of our HD patients had adynamic bone disease [30,31,32,33]. It should not be overlooked that a smaller percentage of HD patients studied here (11%) had iPTH levels higher than 500 pg/mL and were suspected to have secondary hyperparathyroidism, meaning that half of HD patients have some disturbance in the iPH level. It is well known that elevated iPTH levels leading to high-turnover bone disease and low PTH levels, which are risk factors for low bone turnover (adynamic bone disease), can augment VC development and progression [34,35]. When bone turnover is low, as with adynamic bone, the amount of interchangeable calcium and phosphate is decreased, and higher blood concentrations are associated with its intake. Also, bone resorption is more prominent than bone formation, interfering with the buffering function of the skeleton for extra phosphate [36,37]. This phenomenon, known as “calcification paradox”, indicates a high risk of ectopic calcifications, including VC in patients with CKD and dialysis during reduced bone mineralization [38]. By contrast, when high bone turnover is present as in secondary hyperparathyroidism, phosphate is released from the bone, and the reservoir function of the skeleton is destroyed again [5].

In dialysis patients with VC, the interplay between iPTH, FGF 23, and sclerostin is complex. Each biomarker is independently associated with VC, but their interaction appears to potentiate VC. Secondary hyperparathyroidism in CKD contributes to increased bone turnover by releasing calcium and phosphate, which can contribute to VC and indirectly influence FGF 23 and sclerostin regulation. Conversely, low iPTH levels, whether induced by drugs, vitamin D, or parathyroidectomy, may result in elevated levels of FGF 23 and sclerostin [39,40]. Furthermore, recent analyses have shown that increased sclerostin levels seem to reflect slower bone turnover, which is often associated with lower iPTH levels. In contrast, lower plasma levels of sclerostin are linked to vitamin D deficiency and effective phosphate control in patients undergoing HD [41]. These observations underscore the importance of monitoring and managing the aforementioned biomarkers in clinical practice to prevent complications such as VC and bone abnormalities, prompting further exploration of their complex interactions and potential implications for therapeutic interventions.

### 4.2. Other Risk Factors for VC in Pre-HD and HD Group of Patients

Besides the imbalances in biomarkers, some demographic and laboratory data were associated with VC in the examined groups. The patients differed in age in favor of the pre-HD group, and older age was a predictor of VC. In the pre-HD group, a higher prevalence of VC was found in men, which is consistent with some studies in patients with CKD [42]. According to previously published data, potential contributors to the observed gender-related differences involve hormonal, lifestyle, genetic, disease duration factors, pericardial/total fat, lipid profile, inflammatory status, variations in matrix Gla protein (MGP), soluble Klotho, vitamin D, sclerostin, iPTH, FGF 23, and osteoprotegerin levels [42]. In addition, serum ferritin level was independently associated with VC in the pre-HD group. This finding is in line with a previous report that ferritin prevents calcification and osteoblastic transformation of muscle cells [43]. Since higher serum ferritin levels have a protective effect on VC, regular monitoring of ferritin levels in pre-HD patients should be considered. Our study also showed that vitamin D therapy had a protective effect against VC in the pre-HD group. More than 60% of the pre-HD patients had vitamin D deficiency/insufficiency, and one-third of them received vitamin D supplementation. Vitamin D supplementation has an adjunctive role in regressing proteinuria, reversing renal osteodystrophy, and restoring calcification inhibitors in patients with CKD [44]. Therefore, it is advisable to monitor and restore vitamin D deficiency in CKD patients.

The present analysis did not show an independent association between serum phosphate and VC in either pre-HD or HD patients, which has been cited in numerous studies. To comply with the KDIGO recommendations to control phosphate within a target range to minimize complications such as VC and bone disease [13,45], we prescribed regular HD to our patients, as well as a restrictive phosphate diet, and 94.2% of HD patients took calcium-based phosphate binders, either alone or in combination with vitamin D. Thus, most of the patients studied here had normal phosphate levels, and up to 27% had hyperphosphatemia. Nevertheless, strict control of phosphate and calcium levels is still an obligation of nephrologists to prevent VC and cardiovascular diseases.

### 4.3. Significance and Limitations of the Study

Our study showed that VCs are associated with different biomarkers in pre-HD and HD patient groups. The decrease in inhibitors and increase in VC promoters in the HD group led us to speculate on potential avenues for considering interventions, including risk stratification, assessment of treatment response, individualized treatment planning, and contribution to drug research and development. It is important to acknowledge several limitations that warrant consideration when interpreting these results. Our study was constrained by its retrospective nature, limitations in data collection, and potential bias. The cross-sectional design of the study restricted our ability to establish temporal relationships between variables and better elucidate the dynamic interactions between risk factors, biomarkers, and the development of VC over time. The relatively modest sample size and patient heterogeneity in both groups limit the generalizability of our findings, especially in the pre-HD group causing an inference bias. While the present study identified associations of VC, it does not establish causation and emphasize the importance of individualizing therapy.

## 5. Conclusions

Our study showed that biomarkers could be measurable indicators of biological processes underlying VC. Decreases in inhibitors and increases in promoters of VC may serve as a potential guide for considering personalized therapeutic approaches. However, the complex interplay of multiple factors may contribute to VC, and further studies are needed to elucidate the underlying pathways.

## Figures and Tables

**Table 1:** Basal clinical characteristics, presence of co-morbidities, and treatment of the patients in pre-HD and HD groups.

	Pre-HD (*N* = 30)	HD (*N* = 85)	*p*
Demographics			
Age, years	66.7 ± 14.96	57.72 ± 13.07	0.023
Sex, m/f	19/11	40/45	0.141
Underlying kidney disease: (%)			
GN	3 (10)	15 (17.6)	0.394
Nephroangiosclerosis	6 (20)	14 (16.5)	0.779
ADPKD	2 (6.7)	8 (9.4)	1.000
T2DM	5 (9.4)	8 (9.4)	0.442
Nephrolithiasis	1 (3.3)	12 (14.1)	0.178
Others	13 (43.3)	28 (32.9)	0.376
Co-morbidities, yes (%)			
T2DM	4 (13.3)	14 (16.4)	0.778
Hypertension	27 (90)	65 (76.7)	0.029
CVD	14 (46.7)	34 (34.1)	0.527
CVI	2 (6.7)	10 (10.6)	0.728
tumor	1 (3.3)	8 (8.2)	0.442
Treatment, no (%)			
ESA	7 (23.3)	36 (64.3)	0.080
Phosphate binder	21 (70)	80 (94.1)	0.001
Alpha D3	9 (30)	25 (29.4)	1.000
Antihypertensive	16 (53.3)	62 (72.9)	0.068
VC score, no (%)			
0	16 (53.3)	36 (42.35)	0.393
1–2	4 (13.3)	18 (21.2)	0.384
≥3	10 (33.3)	31 (37.47)	0.756
≥6 (8)	1 (0)	15 (6)	0.030

Continuous normally distributed variables are presented as mean ± SD; continuous skewed variables are presented as median (IQR). m = male, f = female, GN = glomerular disease, ADPKD = adult dominant polycystic kidney disease, T2DM = diabetes mellitus type 2, tumor = benign and malignant prostate tumor, hepatocellular liver cancer, bronchial carcinoma, urinary bladder cancer. ESA—erythropoietin stimulating agents, CVD—cardiovascular diseases (pre-study period: heart failure, previous myocardial infarction, aorto-coronary bypass surgery, peripheral vascular disease), CVI—cerebrovascular insult, VC = vascular calcification.

**Table 2:** Basal laboratory data.

	Pre-HD (*N* = 30)	HD (*N* = 85)	*p*
Cholesterol, mmol/L	4.83 ± 1.32	4.59 ± 1.24	0.109
Triglyceride, mmol/L	1.4 (0.8)	1.65 (1.2–2.8)	0.804
Hemoglobin, g/L	106 (96.7–114.5)	108 (101.5–116.5)	0.384
Glycaemia, mmol/L	5.2 (4.7–5.55)	4.9 (4.2–6.05)	0.663
Creatinine, µmol/L	552.5 (318–731.2)	808 (705.5–915)	0.000
Urea, mmol/L	23.4 (14.2–32.95)	21.0 (17.9–26.0)	0.327
Urate, µmol/L	386.70 ± 99.1	362.1 ± 88.9	0.201
Sodium, mmol/L	139.87 ± 3.96	137.86 ± 2.98	0.022
Potassium, mmol/L	4.85 ± 0.80	5.29 ± 0.69	0.008
Calcium, mmol/L	2.16 (2.05–2.28)	2.19 (2.1–2.31)	0.541
Phosphate, mmol/L	1.53 (1.1–1.8)	1.65 (1.3–2.03)	0.247
Ferritin, µg/L	84.3 (50.75–231.87)	355 (131.7–490.1)	0.000
Alkaline phosphatase, IU/L	73.5 (61.5–99.2)	87 (60–109)	0.304
Ln (iPTH), pg/mL	4.97 ± 1.0	4.61 ± 1.37	0.279
<100 *	11 (36.6%)	36 (42.3%)	
25(OH)D, ng/mL	24.7 (46.22)	27.5 (19.2–48.05)	0.879
<29 * (deficiency + insufficiency)	19 (64.28)	45 (53.6%)	
Ln (Fetuin A), ng/mL	6.24 ± 0.25	5.85 ± 0.43	0.001
Ln (Sclerostin), pg/mL	8.12 ± 0.36	7.54 ± 0.91	0.012
Ln (FGF 23), pg/mL	5.01 ± 0.75	6.48 ± 1.11	0.000

All variables are presented as mean ± SD, and median (IQR). * number of patients with percentage in parenthesis. iPTH = intact parathyroid hormone, 25(OH)D = 25-hydroxyvitamin D (deficiency < 20 ng/mL, insufficiency 21–29 ng/mL), FGF 23 = fibroblast growth factor 23.

**Table 3:** Differences between patients with and without vascular calcification within each group.

	Pre HD Group		HD Group		*p*
	1 VC +	2 VC Neg	3 VC +	4 VC Neg	
Number	14	16	49	36	
Age, years	71.7 ± 7.96	62.5 ± 18.98	58.66 ± 13.24	56.43 ± 13.07	1 vs. 3, 4, *p* < 0.001
Sex, m/f	12/2	7/9	23/26	17/19	1 vs. 2, 3, 4, *p* < 0.02
ESA, yes/no	2/12	5/11	20/29	16/20	1 vs. 4, *p* = 0.05
Phosphate binder, yes/no	9/5	12/4	47/2	33/3	1 vs. 3, 4, *p* < 0.03 2 vs. 3, *p* = 0.028
Phosphate, mmol/L	1.57 ± 0.68	1.51 ± 0.33	1.54 ± 0.38	1.87 ± 0.52	4 vs. 2, 3, *p* < 0.018
Phosphate range, mmol/L <1.0 1.0–1.8 ≥1.9	2 8 4 *	2 12 2 *	2 37 ^a^ 10 ^b,^*	0 18 ^a^ 18 ^b,^*	^a^ *p* = 0.020 ^b^ *p*= 0.005 * 2 + 4 vs. 1 + 3, *p* = 0.057
Ln(FGF 23), pg/mL	6.31 (5.98–6.40)	6.26 (6.14–6.30)	5.83 (5.62–6.17)	5.95 (5.46–6.10)	2 vs. 3, 4, *p* < 0.0001 1 vs. 3, 4, *p* < 0.004
Ln(Fetuin A), ng/mL	4.67 (4.17–5.57)	5.13 (4.31–5.60)	6.92 (5.91–7.31)	7.16 (5.85–7.31)	1 vs. 3, 4, *p* = 0.001 2 vs. 3, 4, *p* < 0.003

Continuous normally distributed variables are presented as mean ± SD; continuous skewed variables are presented as median (IQR). m = male, f = female, ESA = erythropoietin stimulating agents, FGF 23 = fibroblast growth factor 23. * means lower number of patients with VC in both subgroups had hyperphosphatemia than patients without VC, ^a^ in HD group with VC more patients had normal serum phosphate, ^b^ in HD group more patients without VC had hyperphosphatemia.

**Table 4:** Predictors of vascular calcification in pre-hemodialysis studied groups, selected with univariate and multivariable linear regression analysis.

	Univariable		Multivariable	
Variables	Standardized Coefficients (95% CI)	*p*	Standardized Coefficients (95% CI)	*p*
Ln (Fetuin A), ng/mL	−0.249 (−7.037–−1.858)	0.120	−0.701 (−0.030–−0.024)	0.001
Ln (Sclerostin), pg/mL	0.590 (1.364–5.651)	0.003	0.970 (4.222–5.764)	0.001
Ln (FGF 23), pg/mL	0.343 (−0.233–2.277)	0.103		
Gender	−0.409 (−3.101–−0.229)	0.025	−1.607 (−8.128–−6.803)	0.000
Age	0.309 (−0.008–0.089)	0.096	1.269 (0.148–0.177)	0.000
Diagnosis	−0.137 (−0.526–0.249)	0.471		
Hemoglobin, g/L	−0.489 (−0.123–0.002)	0.057		
s-creatinine, µmol/L	−0.023 (−0.004–0.004)	0.935		
s-urea, mmol/L	−0.238 (−0.139–0.056)	0.391		
s-urate, µmol/L	0.010 (−0.007–0.007)	0.963		
s-calcium, mmol/L	−0.075 (−6.067–4.848)	0.812		
s-phosphate, mmol/L	0.012 (−1.866–1.957)	0.961		
Alk. Phosphatase, IU/L	0.085 (−0.014–0.020)	0.681		
Ln (iPTH), pg/mL	−0.456 (−0.005–0.004)	0.030		
Ferritin, µg/L	−0.241 (−0.012–0.003)	0.083	−0.252 (−0.007–−0.002)	0.014
Phosphate binder	−0.097 (−2.060–0.788)	0.612		
Vit D treatment	−0.177 (−2.247–0.796)	0.104	−0.187 (−1.259–−0.274)	0.022

iPTH = intact parathyroid hormone, FGF 23 = fibroblast growth factor 23, Diagnosis = diagnosis of cardiovascular diseases.

**Table 5:** Predictors of vascular calcification in the hemodialysis group, selected with univariate and multivariable linear regression analysis.

	Univariable		Multivariable	
Variables	Standardized Coefficient (95% CI)	*p*	Standardized Coefficient (95% CI)	*p*
Ferritin, µg/L	0.014 (−0.002–0.002)	0.934		
(Ln) Fetuin A, ng/mL	−0.238 (−4.446–1.331)	0.280		
(Ln) Sclerostin, pg/mL	0.322 (0.333–2.247)	0.102	0.902 (1.967–3.542)	0.001
(Ln) FGF 23, pg/mL	−0.105 (−0.407–0.003)	0.109	0.593 (1.216–2.568)	0.001
Gender, male	0.089 (−0.911–1.862)	0.495		
Age	−0.008 (−0.106–0.102)	0.971		
dialysis vintage, months	−0.018 (−0.009–0.008)	0.892		
Diagnosis	0.195 (−0.296–0.776)	0.359		
s-cholesterol, mmol/L	0.697 (−0.005–2.640)	0.051		
Hemoglobin, g/L	−0.286 (−0.146–0.025)	0.156		
s-creatinine, µmol/L	0.065 (−0.002–0.004)	0.618		
s-urea, mmol/L	0.565 (−0.001–0.491)	0.051	0.446 (0.069–0.318)	0.004
s-urate, µmol/L	−0.066 (−0.010–0.006)	0.614		
s-calcium, mmol/L	0.019 (−3.817–4.42)	0.884		
s-phosphate, mmol/L	0.035 (−0.976–1.247)	0.807		
Alk. Phosphatase, IU/L	−0.046 (−0.018–0.013)	0.727		
(Ln) iPTH, pg/mL	−0.080 (−0.419–−0.246)	0.058	−0.895 (−1.049–−0.008)	0.001
Phosphate binder	0.013 (−3.051–3.384)	0.918		
Vit. D treatment	0.118 (−0.837–2.246)	0.364		

iPTH = intact parathyroid hormone, FGF 23 = fibroblast growth factor 23, Diagnosis = diagnosis of cardiovascular diseases.

## Data Availability

All data generated or analyzed during this study are included in this article. Further inquiries can be directed to the corresponding author.
